# Germline and somatic polymerase ε and δ mutations define a new class of hypermutated colorectal and endometrial cancers

**DOI:** 10.1002/path.4185

**Published:** 2013-05-13

**Authors:** Sarah Briggs, Ian Tomlinson

**Affiliations:** 1 Molecular and Population Genetics Laboratory, Wellcome Trust Centre for Human Genetics, University of OxfordRoosevelt Drive, Oxford, OX3 7BN, UK; 2 Oxford NIHR Comprehensive Biomedical Research Centre, Wellcome Trust Centre for Human Genetics, University of Oxford, Roosevelt DriveOxford, OX3 7BN, UK

**Keywords:** DNA polymerase, exonuclease, proofreading, germline mutation, somatic mutation, colorectal cancer, colorectal adenomas, polyposis, endometrial cancer

## Abstract

Polymerases ϵ and δ are the main enzymes that replicate eukaryotic DNA. Accurate replication occurs through Watson–Crick base pairing and also through the action of the polymerases' exonuclease (proofreading) domains. We have recently shown that germline exonuclease domain mutations (EDMs) of *POLE* and *POLD1* confer a high risk of multiple colorectal adenomas and carcinoma (CRC). *POLD1* mutations also predispose to endometrial cancer (EC). These mutations are associated with high penetrance and dominant inheritance, although the phenotype can be variable. We have named the condition polymerase proofreading-associated polyposis (PPAP). Somatic *POLE* EDMs have also been found in sporadic CRCs and ECs, although very few somatic *POLD1* EDMs have been detected. Both the germline and the somatic DNA polymerase EDMs cause an ‘ultramutated’, apparently microsatellite-stable, type of cancer, sometimes leading to over a million base substitutions per tumour. Here, we present the evidence for *POLE* and *POLD1* as important contributors to the pathogenesis of CRC and EC, and highlight some of the key questions in this emerging field. Copyright © 2013 Pathological Society of Great Britain and Ireland. Published by John Wiley & Sons, Ltd

## Introduction

In comparison with other cancers, there exist a relatively large number of syndromes in which a high lifetime risk of colorectal cancer (CRC) is caused by inheriting a mutation in a single gene. The specific Mendelian CRC syndromes (and their mutant genes) are familial adenomatous polyposis (*APC*), Lynch syndrome/HNPCC (mismatch repair genes *MSH2*, *MLH1*, *MSH6*, *PMS2*), Peutz–Jeghers syndrome (*LKB1/STK11*), juvenile polyposis (*SMAD4*, *BMPR1A*), *MUTYH*-associated polyposis (the base excision repair gene *MUTYH*), and hereditary mixed polyposis (*GREM1*). All of these conditions, except *MUTYH*-associated polyposis, are dominantly inherited, although a recessive version of HNPCC exists, in which both copies of one of the mismatch repair (MMR) genes are mutated. Each syndrome differs in its clinical features, but in most cases, there is a primary predisposition to multiple (10s–1000s) adenomas or other benign polyps, with a secondary CRC risk, probably owing to progression of the benign lesions. The exception is Lynch syndrome, in which there is usually a small excess of adenomas and the primary predisposition is to CRC.

An ongoing question for several years has been whether there are any more high-penetrance CRC predisposition genes to be found. There certainly exist patients whose clinical features and family history make them *a priori* likely to carry a high-penetrance CRC predisposition allele, but who have no mutations in the known Mendelian CRC genes. One such group of patients comprises individuals with hyperplastic polyposis syndrome (HPPS), although the hypothetical HPPS gene(s) has not yet been identified. Another set of patients likely to carry high-penetrance CRC mutations has multiple adenomas. Typically, these tumours number 10–100 at presentation or after a few years of screening. Patients may present before the age of 60 and they have often developed one or more CRCs. Some of these individuals come from extensive CRC pedigrees, although others have no significant family history of colorectal tumours.

In this article, we summarize the recent identification of DNA polymerase ϵ and δ mutations in familial colorectal cancer cases, many of whom have multiple adenomas. In common with other genes such as *APC* and *SMAD4*, the polymerases are additionally somatically mutated in a recently-reported subset of sporadic CRCs 1. There is also accruing evidence that, like the mismatch repair genes, *POLE* and *POLD1* mutations play roles in endometrial carcinogenesis.

## High-penetrance germline DNA polymerase ϵ and δ mutations cause colorectal and endometrial cancers

Using a combination of whole-genome sequencing of highly-selected multiple adenoma patients, linkage analysis, and studies of loss of heterozygosity (LOH) in tumours, followed by replication in a large set of familial CRC cases, we recently identified two specific germline mutations that caused carriers to develop multiple colorectal adenomas and CRC 2. These mutations were in two related DNA polymerase genes: *POLE* (p.Leu424Val) and *POLD1* (p.Ser478Asn). Neither mutation was present in nearly 7000 UK controls or in public databases of controls. Subsequently, we found an additional, probably pathogenic mutation, *POLD1* p.Pro327Leu, in a further multiple adenoma patient.

**Figure 1:**
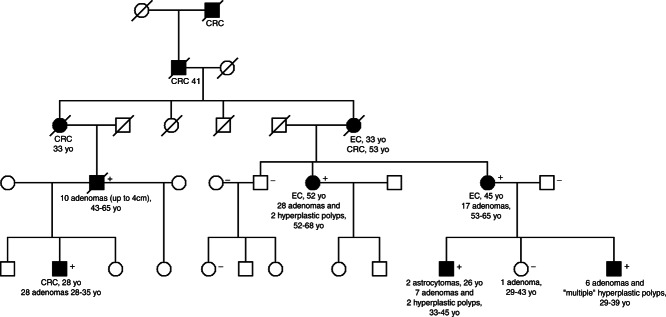
Pedigree of a *POLD1* p.Ser478Asn family. Shading denotes those affected with multiple (> 5) colorectal adenomas (adenomas) and/or early-onset colorectal cancer (CRC). In addition, three women developed endometrial carcinoma (EC). + denotes individuals tested and found to be gene carriers, and − denotes tested non-carriers. Ages denote the time interval over which colorectal polyps developed or the time at which cancer occurred. Note that one non-gene carrier developed a very small colorectal adenoma by age 43 years and that one carrier developed two astrocytomas, raising the possibility that *POLD1* mutations also predispose to this tumour type.

Both mutations show dominant inheritance and confer high-penetrance predisposition to multiple colorectal adenomas, large adenomas, early-onset CRC, or multiple CRCs (Figure 1). For this reason, we have called the disease polymerase proofreading-associated polyposis (PPAP). The phenotype varies among carriers: some have tens of adenomas that do not always appear to progress rapidly to cancer, whereas others have a small number of large adenomas or carcinomas. The histological features of the tumours are unremarkable. They are mostly conventional adenomas and carcinomas that occur throughout the large bowel. There is currently no evidence that mutation carriers are at risk of upper-gastrointestinal tumours, but female carriers of *POLD1* p.Ser478Asn have a greatly increased risk of endometrial cancer (EC). Overall, the PPAP phenotype overlaps with the phenotypes associated with germline mutations in *APC*, *MUTYH*, and the MMR genes. *POLE* p.Leu424Val, *POLD1* p.Ser478Asn, and *POLD1* p.Pro327Leu all map within the proofreading (exonuclease) domain of the respective enzyme, suggesting that deficient proofreading repair during DNA replication is the cause of our patients' tumours. It is of note that POLD1 in particular additionally participates in both MMR and base excision repair. However, the families' tumours are microsatellite-stable. Although the reasons for this are currently unclear, it is possible to speculate that the strand slippage that results in insertion–deletion mutations does not involve a mispaired, single-strand intermediate that is recognized by the polymerase proofreading domain, and, moreover, that polymerase proofreading has a minor role in mismatch repair.

## Somatic polymerase ϵ mutations in sporadic colorectal and endometrial cancers

Separately from the work that discovered germline *POLE* and *POLD1* mutations in colorectal cancer patients, *POLE* was highlighted as a somatically mutated gene in CRC by The Cancer Genome Atlas (TCGA) exome sequencing project 1. A set of cancers with a very large number of coding mutations (over 50 per 10^6^ bases), in the absence of MMR defects or microsatellite instability (MSI), was also identified, and this set of cancers overlapped with the *POLE*-mutant set. Subsequently, it was found that almost all of the hypermutant, MS-stable cancers had *POLE* exonuclease domain mutations (EDMs) 2,3. The seven *POLE* EDMs in the TCGA cohort, out of a total of 226 CRCs (3%), were all missense changes, although the germline p.Leu424Val change was absent. Two recurrent changes were found, p.Val411Leu and p.Ser459Phe. These data are consistent with those from another CRC exome sequencing project 4 that found two of 74 (3%) cancers to have acquired *POLE* p.Pro286Arg mutations. Recently released, but unpublished data from the TCGA have confirmed codons 286, 411, and 459 as mutation hotspots 5. Interestingly, *POLE* residue 286 is homologous to the probably pathogenic germline mutation that we have reported at residue 327 in *POLD1*. However, there is no good evidence of pathogenic, somatic *POLD1* EDMs.

**Figure 2:**
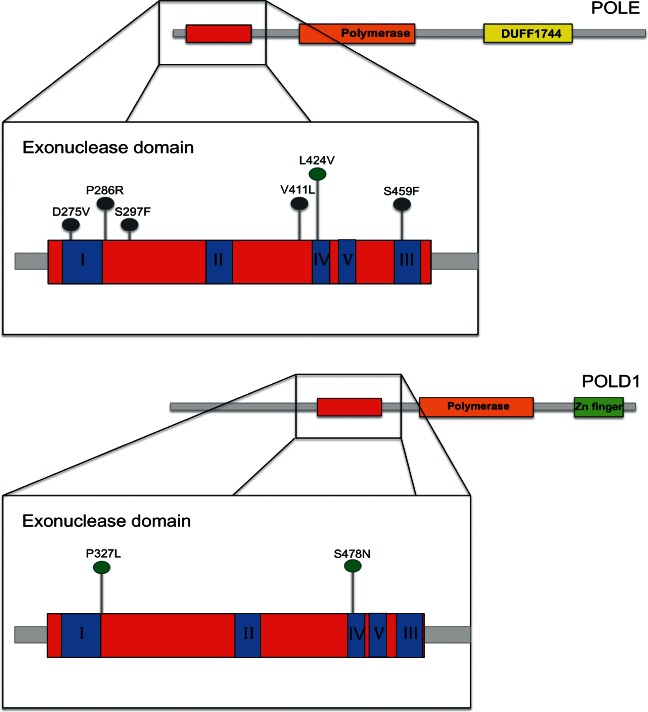
The structure of POLE and POLD1 demonstrating the position of key mutations. Conserved exo motifs I–V within the exonuclease domain are highlighted in blue. Green circles denote germline mutations; grey circles denote somatic mutations.

The TCGA 5 and we ourselves 6 have found somatic *POLE* EDMs in ECs. These occur at a slightly higher frequency (∼7%) than that in CRCs. As was the case for CRCs, *POLD1* EDMs were very rare common, seen in just one tumour in each cohort (approximately 0.5%). The *POLE* mutation spectra of the ECs showed overlap with those of the CRCs, with p.Pro286Arg and p.Val411Leu particularly frequent. p.Ser297Phe was also found in two ECs. In addition, a somatic p.Leu424Val change – the mutation present in the germline of CRC cases – was present in two ECs, and one cancer possessed a mutation of a residue that forms the exonuclease catalytic site (p.Asp275Val) (Figure 2). Like CRCs, the *POLE*-mutant ECs tended to be MSI-negative and had an ultramutator phenotype. Figure 2 shows the locations of the somatic and germline *POLE* and *POLD1* mutations within the exonuclease domain.

## The normal roles of polymerase ϵ and δ

POLE and POLD1 are related B family polymerases. They form the major catalytic and proofreading subunits of the Polϵ and Polδ enzyme complexes that respectively synthesize the leading and lagging strands in DNA replication 7,8. Their proofreading (exonuclease) function detects and removes misincorporated bases in the daughter strand through failed complementary pairing with the parental strand. The high fidelity of DNA replication is in part due to very low error rates in dNTP incorporation by the polymerase (10^−4^ to 10^−5^) and in part due to proofreading by the exonuclease domain, which improves this fidelity approximately 100-fold. *POLE* and *POLD1* have greatest homology (23% identity, 37% similarity) over their exonuclease domains (residues 268–471 of *POLE* and 304–517 of *POLD1*). Both genes are ubiquitously expressed and show high levels of evolutionary conservation, especially in the exonuclease domain. A number of yeast mutants exist in the homologues of *POLE* and *POLD1*, and these models have shown that mutator phenotypes can result from outside in the polymerase domain 9–14. Other variants actually have improved DNA repair capacity 15, although they also have lower processivity (rate of synthesis of the daughter strand).

The Polϵ and Polδ enzymes are both heterotetramers in higher eukaryotes. The accessory subunits (POLE2/3/4 and POLD2/3/4) are involved in regulating synthesis and in binding co-factors such as PCNA. It is of note that a common polymorphism within *POLD3* has been found to be associated with a modestly increased risk of CRC in the general northern European population 16. Whether this acts in a similar way to *POLD1* mutations is currently unclear.

POLD1 – and perhaps also POLE – is thought to play an additional role in new strand synthesis as part of the processes of base excision repair and MMR. POLE is involved in break-induced replication, a form of double-strand break repair in which the homologous chromosome is used as a template, resulting in copy-neutral LOH. Whether these aspects of DNA polymerase function are important for tumour development is unknown, as is the explanation for the near-absence of somatic *POLD1* EDMs. At the very least, it is striking that defects in at least three pathways involved in the repair of base pair-level mutations can predispose to colorectal tumours.

## How do *POLE* and *POLD1* mutations cause tumorigenesis?

**Figure 3:**
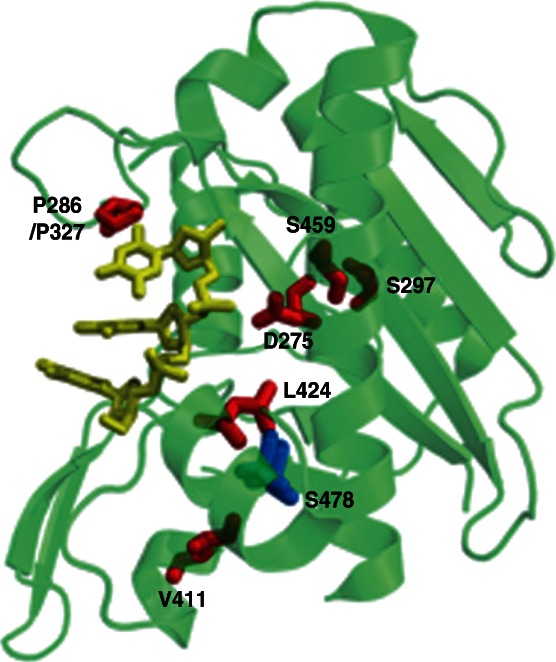
Pymol-generated image of *POLE* and *POLD1* EDMs on a composite structure of yeast Polδ (PDB 3IAY) and the ssDNA component (yellow) of the T4 polymerase complex (PDB 1NOY). Mutant amino acids are shown in red (POLE) or blue (POLD1). The key mutations generally cluster around the active site (D275) close to the ssDNA, an exception being V411 which lies some distance away and may act through affecting the positions of other residues closer to the active site.

The most common somatic and germline mutations in *POLE* and *POLD1* have been mapped onto a hybrid structure of yeast DNA polymerase (3iay) and T4 polymerase (Figure 3). The residues equivalent to the two germline mutations (*POLE* p.Leu424Val and *POLD1* p.Ser478Asn) pack together at the interface between two helices that form the base of the exonuclease active site. Mutations are predicted to distort the packing of the helices; this will in turn be propagated to the active site, affecting nuclease activity 2. The residue equivalent to the most common somatic *POLE* mutation at amino acid 286 localizes to the DNA binding pocket adjacent to the exonuclease active site, with its side chain very close to the nascent single-stranded DNA, and substitutions at this site are predicted significantly to perturb the structure of the DNA binding pocket. Structural analysis also shows that amino acid 297 interacts with exonuclease catalytic site residue 275, and mutation here would probably alter the active site conformation. Interestingly, *POLE* residue 411, whilst conserved, is not predicted to interact with DNA or catalytic site residues, and the effects on tumorigenesis may be through secondary effects on the binding pocket. Although the structural data do not explain the recurrent nature of some *POLE* mutations, they strongly suggest that the *POLE* and *POLD1* mutations impair polymerase proofreading. For most of these mutations, moreover, studies in model organisms including T4 bacteriophage, yeast, and mice have confirmed these effects 2 #path4185-bib-0010 #path4185-bib-0017"/>. For example, the p.Pro123Leu mutation in the T4 bacteriophage is at the equivalent residue to human Pro286 and produces a strong mutator phenotype 18.

Hypermutation is thus a very plausible consequence of EDM *POLE* and *POLD1* mutations. Whether this is the only tumour-promoting consequence of these mutations remains unclear. It will also be intriguing to determine whether proofreading deficiency has any effect on polymerase processivity, since negative effects on this function may be selectively deleterious for the cell.

## Pathways of tumorigenesis

Exome sequencing data from CRCs and ECs with somatic *POLE* EDMs show that the coding regions alone of these tumours have acquired a mean of about 5000 somatic base substitutions 1,5. All types of base substitution are increased in frequency compared with cancers without EDMs, and C:G → T:A changes generally remain the most common. However, the mutation spectrum is changed, with a particular increase in the proportion of G:C → T:A and A:T → C:G transversions. Although there is considerable variation in the number of mutations among cancers with EDMs, there is some evidence that specific mutations have different effects on the mutation spectrum. For example, cancers that carry p.Pro286Arg show a much stronger bias towards transversions than cancers with p.Val411Leu. Interestingly, the exome sequence data show that the somatic mutations resulting from deficient exonuclease proofreading tend to occur at sites flanked by an A base on the positive DNA strand, rather than by T, G or C. The causes for this observation are currently unknown, although one possibility is the ability of mutations to be corrected by the MMR machinery.

*POLE* and *POLD1* are not classical tumour suppressor genes, as loss-of-function mutations appear not to be pathogenic. Instead, there is loss of a specific function, proofreading, that is unlikely to be achieved through protein-truncating mutations. Furthermore, it is not clear whether ‘two hits’ at *POLE* or *POLD1* are required for tumourigenesis. In *Pole*-mutant mice, a mutator phenotype and increased frequency of tumour formation are only seen when *Pole* mutations are homozygous 17. In humans, some, but not all, tumours from patients with germline *POLE* or *POLD1* mutations show LOH, although data on other forms of ‘second hit’ are lacking in these tumours. LOH has not been reported in cancers with somatic *POLE* mutations, although a few of these tumours have protein-truncating mutations that could act as ‘second hits’. From a functional perspective, a Polϵ or Polδ protein with a heterozygous EDM would probably cause an increase in mutation frequency because 50% of polymerase activity would be error-prone. However, it is conceivable that this error rate is insufficient to overwhelm the other repair systems such as MMR.

A further unanswered question is when *POLE* and *POLD1* mutations occur during tumourigenesis. For colorectal tumours with either somatic or germline *POLE* and *POLD1* EDMs, the mutation spectrum of the *APC* gene shows deficiency of frameshift (insertion–deletion) changes compared with other CRCs. Moreover, some *APC* mutations that are generally rare in CRCs are relatively common in *POLE*- and *POLD1*-mutant tumours, an example being the Arg1114X change. These data suggest that the *POLE* and *POLD1* mutations occur before *APC* mutations. However, at least in sporadic CRCs with *POLE* EDMs, the frequency of pathogenic mutations in the other known driver genes is low, perhaps suggesting that they follow an atypical pathway of tumorigenesis.

## *POLE* and *POLD1* mutations outside the exonuclease domain

*POLE* and *POLD1* are both quite large genes, with protein-coding regions of about 6.6 and 3.3 kb, respectively. Inevitably, therefore, they will acquire many ‘passenger’ mutations in cancers. The causal role of EDMs has been deduced as a result of finding (i) germline mutations in PPAP and (ii) the so-called ‘ultramutator’, MSI-negative phenotype in sporadic cancers. No such clues exist in support of a pathogenic role for the non-exonuclease domain *POLE* and *POLD1* mutations that are found in 3–4% of CRCs and ECs. In fact, the majority of these non-EDM changes occur in MSI-positive cancers and most seem likely to be passengers.

## Future prospects

Evidently, the identification of germline and somatic *POLE* and *POLD1* mutations that cause CRC and EC is only the first stage in understanding how those changes act and, if possible, exploiting them for cancer prevention and therapy. Perhaps the immediate priority is to determine the full Polϵ and Polδ mutation spectra, to determine which mutations are pathogenic, and then to understand their effects. The somatic mutation spectrum of *POLE* and *POLD1* in the common cancers will be increasingly well described in the coming months as further large-scale sequencing programmes come to fruition. It will also be important to test *POLE* as a prognostic marker. In the germline, similar mutation profiling efforts will undoubtedly be performed, one scenario being that there exist a number of germline Polϵ and Polδ variants with different magnitudes of effect on risk. Given that common polymorphisms have been addressed by CRC and EC GWAS, the uncharacterized germline risk variants are likely to be individually uncommon (<5% allele frequency) and some may not necessarily be in the exonuclease domains of *POLE* and *POLD1*. It will be necessary to obtain evidence for their effects using a variety of functional assays. A particularly interesting issue is why germline *POLE* or *POLD1* mutations can cause cancer, yet only *POLE* is somatically mutated. This has a parallel in the MMR genes, of which four can be mutated in Lynch syndrome, but only *MLH1* plays a role somatically. It may be that *POLD1* has an as yet unidentified essential function that precludes its somatic mutation or that the effects of somatic mutation are too weak (or indeed too strong) to be effective other than in the germline setting. Finally, the discovery of a new type of CRC and EC based on *POLE* mutations rather than the established classifiers of MSI and chromosomal instability raises the prospect of future similar discoveries, leading to an increasingly refined classification of cancer based on DNA analysis, which is potentially more robust than analysis of gene or protein expression.
